# Leisure-Time Physical Activity Interventions for Health Promotion in Individuals With Rett Syndrome: Scoping Review

**DOI:** 10.2196/91550

**Published:** 2026-09-21

**Authors:** Christen John Mendonca, James H Rimmer, Ashley Wright, Raven Young, Amitha Ananth, Byron Lai

**Affiliations:** 1 SHP Research Collaborative School of Health Professions University of Alabama at Birmingham Birmingham, AL United States; 2 Dean’s Office, School of Health Professions University of Alabama at Birmingham Birmingham, AL United States; 3 Division of Pediatric Rehabilitation Medicine University of Alabama at Birmingham Birmingham, AL United States; 4 Heersink School of Medicine Division of Pediatric Neurology University of Alabama at Birmingham Birmingham, AL United States

**Keywords:** Rett syndrome, physical activity, neurodevelopmental disorder, rehabilitation, telehealth

## Abstract

**Background:**

While half of individuals with Rett syndrome (RTT) are older than 50 years, research shows that they have low levels of physical activity, especially for those of advanced age and poor ambulation. Despite this evidence, recent studies underscore the potential of leisure-time physical activity interventions to improve health outcomes and quality of life for individuals with RTT.

**Objective:**

This scoping review aimed to summarize the state of the science regarding physical activity interventions for individuals with RTT and their families across the life span; map the extent, range, and nature of research activity in telehealth; and describe outcomes targeted by interventions with a focus on health-related fitness.

**Methods:**

Systematic searches were performed in the MEDLINE, Scopus, Google Scholar, and CINAHL Plus databases. The data charted from eligible studies included specific details about the participants, study design, setting, intervention characteristics, outcomes, and relevant key findings. Inclusion criteria were (1) original, peer-reviewed research; (2) full-text articles in English; (3) studies reporting a leisure-time physical activity intervention; and (4) a sample of individuals with RTT and/or caregivers.

**Results:**

A total of 23 studies enrolled 200 participants in total (mean age 13, SD 7, range 2-48 years). Participants were all female individuals, and studies included mostly those who were ambulatory. RTT severity was reported in less than half (9/23, 39%) of the studies; however, participants with mild to severe scores were represented. Most of the studies (20/23, 87%) used a case study design or single-group repeated measures, and only 13% (3/23) were randomized trials. The focus was primarily on gross motor function, walking ability, and physical activity outcomes. In total, 39% (9/23) of the interventions implemented telehealth into their design, primarily using remote video calls between a physical therapist and the participants’ parents.

**Conclusions:**

The findings of this review provide a foundation for designing evidence-based, scalable physical activity programs tailored to the unique needs of individuals with RTT. The integration of telehealth strategies offers a promising avenue for enhancing accessibility and caregiver engagement.

## Introduction

Rett syndrome (RTT) is a rare progressive neurodevelopmental disorder that is prevalent among female individuals [1,2]. RTT is characterized by impairments in motor function, communication, and cognitive abilities [3-5]. Half of individuals with RTT are older than 50 years [6]. Considering that mortality in RTT has been linked with several preventable factors such as declines in physical function and cardiorespiratory issues [7], there is a need to identify interventions that can improve and manage physical health and wellness.

Over 40 years of research have found that leisure-time physical activity (LTPA) participation has been identified as a key behavior for improving and managing physical and mental health among people with mobility disabilities [8,9]. LTPA refers to physical activities performed outside of regular work, including exercise; sports; and physically active hobbies such as walking for exercise, running, and similar activities done during discretionary time [10]. While there is a growing volume of research related to LTPA in more prevalent disability groups (eg, stroke, Parkinson disease, cerebral palsy, and multiple sclerosis) [8,9,11,12], less is known about its potential impact in preserving or improving health in individuals with RTT.

Although there are systematic reviews that highlight the multifaceted benefits of specific rehabilitation interventions in people with RTT [13-18], there has not been a review of LTPA interventions for this population. Compiling a single resource of LTPA interventions for RTT can help health professionals and caregivers quickly identify programs that extend outside the rehabilitation context and promote physical health and wellness across the life span. To provide a foundation that can guide future research in LTPA and telehealth for individuals with RTT, the objectives of this scoping review were as follows:

1. To aggregate and summarize the state of the science regarding LTPA for people with RTT across the lifespan
2. To describe LTPA intervention characteristics and their potential outcomes on physical health, function, and fitness
3. To explore telehealth technologies and their use in LTPA interventions

## Methods

### Study Design

This scoping review was conducted using the methodological framework developed by Arksey and O’Malley [19], which consists of (1) identifying a research question; (2) identifying relevant studies; (3) selecting studies; (4) charting the data; (5) collating, summarizing, and reporting the results; and (6) an optional consultation exercise. Additionally, recommendations and considerations to improve scoping reviews in health research were implemented [20,21]. This research adhered to the PRISMA (Preferred Reporting Items for Systematic Reviews and Meta-Analyses) guidelines for reporting, and a completed PRISMA-ScR (Preferred Reporting Items for Systematic Reviews and Meta-Analyses Extension for Scoping Reviews) checklist can be found in Multimedia Appendix 1.

### Review Questions

The population, intervention, comparison, and outcome framework [22] was used to develop the guiding questions for this scoping review: (1) What is the state of the science regarding LTPA interventions for individuals with RTT? (2) What are the intervention delivery strategies and their outcomes, with a specific focus on health-related fitness [23] (cardiorespiratory fitness, muscular strength, muscular endurance, body composition, and flexibility)? (3) What telehealth strategies have been implemented?

### Inclusion Criteria

Studies were deemed eligible for inclusion if they met the following criteria: (1) publications that were primary sources of original, peer-reviewed research; (2) LTPA interventions that incorporated the deliberate use of repetitive large body movements to improve, maintain, or change a component of health (exercise, recreation, and conditioning); (3) interventions that targeted people with RTT and/or caregivers; and (4) LTPA interventions that could be delivered in the community outside a rehabilitation setting and did not require the supervision of a licensed therapist. This study included all types of research designs, from case studies to randomized controlled trials (RCTs). Gray literature articles were excluded from review.

### Search Process

A preliminary search of the PubMed database, Cochrane Database of Systematic Reviews, and Scopus database was conducted (CM), and no current or underway systematic or scoping reviews on the topic were identified. The text words contained in the titles and abstracts of relevant articles and the index terms used to describe the articles were used to develop a full search strategy.

The article search was conducted (CM) for all years up to September 30, 2025, in the following databases: MEDLINE, Scopus, and CINAHL Plus. Hand searches also included research found through Google Scholar and from the reference lists of existing RTT reviews.

### Search Strategy

Article searches were generally conducted by integrating RTT terms (eg, “Rett syndrome” and “neurodevelopmental disorder”) and LTPA key terms (eg, “physical activity,” “exercise,” and “sedentary activity”). Boolean operators were used to connect RTT terms with physical activity terms depending on the database. The MEDLINE database search string was as follows: “Rett syndrome AND physical activity.”

### Study and Source of Evidence Selection

Following the search, all identified citations were collated and uploaded into EndNote 2025 (Clarivate Analytics), and duplicates were removed. Titles and abstracts were screened by 2 independent reviewers (CM and BL) for assessment against the inclusion criteria for the review. Potentially relevant sources were retrieved in full text and saved as a PDF to a shared digital folder. Rationales for the exclusion of articles were recorded. Any disagreements that arose between the reviewers at each stage of the selection process were resolved through discussion with an additional reviewer (JR) during weekly meetings. The results of the search and the study inclusion process are reported in full and presented in a PRISMA flow diagram.

### Data Charting

Data were extracted from the full texts of the articles by 3 reviewers. Extraction was guided by a data template in Microsoft Excel. One reviewer (CM) extracted data from all articles. Two reviewers (RY and AW) each reviewed half of the total articles as a way to ensure that all articles were reviewed by 2 reviewers. After reviewers charted data from the first 2 studies, the research team met to resolve any disputes and aggregate the data extraction results. Extracted data included specific details about the participants, concept, context, study methods, interventions, outcomes, and key findings relevant to the review questions. The final data extraction chart was cross-checked for accuracy by 2 analysts (CM and BL).

### Data Analysis and Presentation

The data were organized graphically and in tabular form. These data included study parameters (design and country of origin), participant demographics and clinical characteristics (age, RTT severity, and ambulatory ability), intervention characteristics (duration, type, frequency, setting, and mode of delivery), outcomes and measures, and telehealth characteristics where applicable (mode of delivery and contact frequency). In instances in which there were multiple publications that were associated with a single trial, the extracted data were considered as a single article for the purposes of summation calculation or frequency counts.

A draft of the preliminary results and findings of this work was reviewed by the mother of an individual with RTT. Consistent with expert recommendations [20], she provided feedback to confirm whether the findings aligned with her experiences, identify any misinterpretations, suggest alternative conceptualizations, and add context to this work. Her feedback was documented in writing through email and integrated into this final work.

## Results

### Search Results

The search strategy yielded 459 records from 122 original research studies. After screening, of the 459 records, 31 (7%) were assessed for eligibility. Of these 31 publications, 8 (26%) were excluded due to not targeting individuals with RTT or caregivers (n=1) [24], not incorporating an LTPA intervention (n=4) [17,25-27], or administering an intervention that required a licensed therapist (n=3) [28-30]. After assessment, 23 articles were included in the final synthesis (Figure 1).

**Figure 1:**
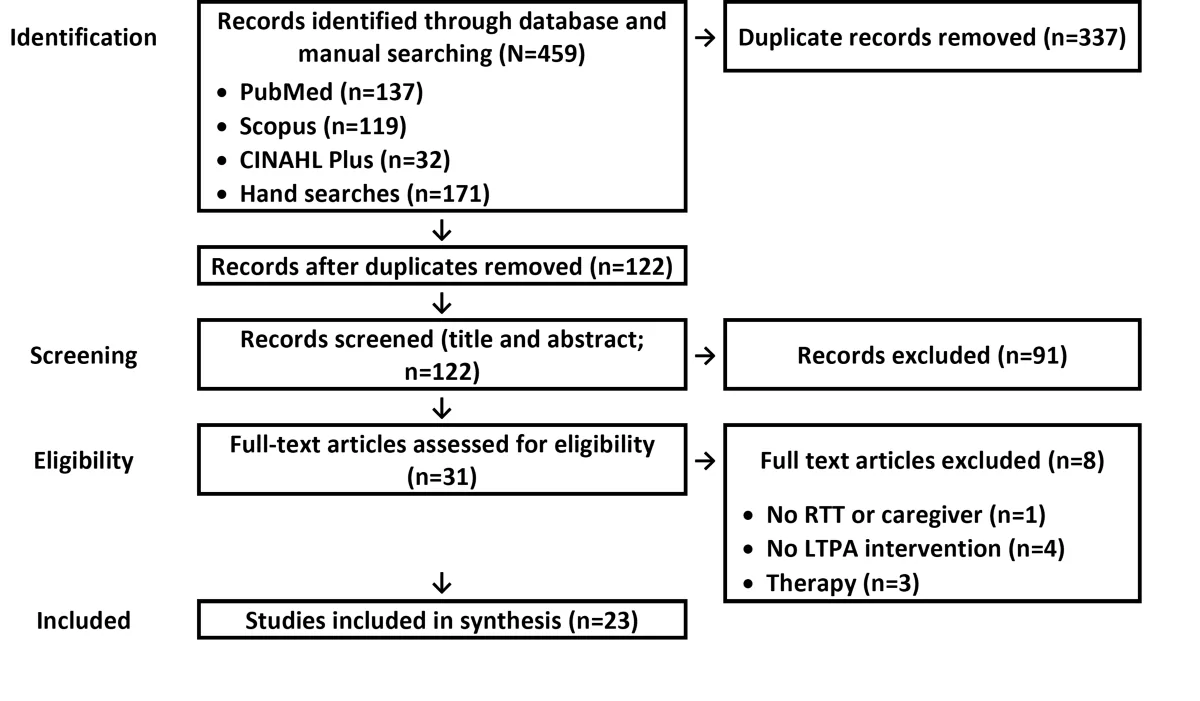
PRISMA (Preferred Reporting Items for Systematic Reviews and Meta-Analyses) flow diagram for the scoping review. LTPA: leisure-time physical activity; RTT: Rett syndrome.

### Study Parameters

General study parameters are shown in Table 1. Most studies (21/23, 91%) were conducted in the last 20 years. The most common research designs were case studies (11/23, 48%) and within-group repeated measures (9/23, 39%). Among the 23 studies, 3 (13%) randomized designs were identified, with only 2 (9%) being RCTs. Almost every study reported at least one favorable result of their interventions, with the most common benefits being improved gross motor function (eg, sitting independently and standing transitions), improved walking ability, or an increase in physical activity or decrease in sedentary time.

**Table 1:** General study parameters.

Study	Year	Design	Country	Notable results
Bumin et al [31]	2003	Case report	Turkey	Hand stereotypies reduced, feeding and hand skills improved, walking balance improved, environmental interaction increased, hyperactive behavior decreased, and anxiety decreased
Schaefer-Campion and Johnson [32]	2015	Case study	United States	Walked furthest using a toy shopping cart and anterior walker compared to other walking aids
Lerma-Castaño et al [33]	2024	Case study	Colombia	Improved gross motor function, balance, obstacle avoidance, and behavior
Downs et al [34]	2018	Randomized controlled trial	China	Improved gross motor function and BMI slightly decreased
Downs et al [35]	2023	Randomized controlled trial	Australia and Denmark	Sedentary time decreased
Imamura et al [36]	2020	Case study	Japan	Improved 10-m walk test performance
Kapel et al [18]	2022	Case study	Slovenia	Improved gross motor function
Lotan et al [37]	2004	Repeated measures	Israel	Improved gross motor function and cardiorespiratory fitness
Lotan and Barmatz [38]	2009	Case study	Israel	Improved communication and bodily control
Lotan et al [39]	2015	Case study	Israel	N/A^a^
Lotan et al [40]	2021	Repeated measures	Italy	Improved motor function, rehabilitation goal achievement, and good parental satisfaction
Lotan et al [41]	2021	Repeated measures	Ireland	Favorable telehealth rating by parents and positive rehabilitation goal attainment
Panzeri et al [42]	2024	Repeated measures	Italy	Improved endurance, participant preference for autonomous activities, and good parental satisfaction
Rocco et al [43]	2023	Case study	United States	Improved participation
Romano et al [44]	2022	Repeated measures	Italy	Most rehabilitation goals attained, improved gross motor function, increased physical activity, and higher motor functioning correlated with higher PA^b^ level
Romano et al [45]	2022	Repeated measures	Italy	Scoliosis progression prevented and improved gross motor function
Romano et al [46]	2022	Repeated measures	Italy	Positive rehabilitation goal achievement; improved gross motor function; and high usefulness, adherence, and satisfaction
Romano et al [47]	2025	Repeated measures	Italy	Improved gross motor function
Stahlhut et al [48]	2020	Repeated measures	Denmark	Intervention rated as feasible; reduced sedentary time; and increased step count, walking capacity, quality of life, and rehabilitation goal attainment
Stasolla and Caffo [49]	2013	Case study	Italy	Increased ambulation and happiness and reduced stereotypies
Stasolla et al [50]	2018	Case study	Italy	Improved functional ability, mood, and social relationships
Escobar Torres et al [51]	2019	Case study	Spain	Improved functional ability, mood, social relationships, mobility, strength, and endurance
Zwilling et al [52]	2022	Randomized between-group intervention	Italy	COVID-19 lockdown did not negatively affect mothers’ well-being

^a^N/A: not applicable.

^b^PA: physical activity.

### Participants

Participant information is shown in Table 2. A total of 200 individuals with RTT were enrolled across the 23 included studies. In total, 13% (3/23) of the publications overlapped and reported separate results from the same sample of participants [40,44,52]. Sample sizes ranged from 1 to 42. Adults were underrepresented, with the mean participant age being 13 (SD 7) years and ages ranging from as young as 2 up to 48 years. A total of 65% (130/200) of the participants were ambulatory (independent or with assistance); 25% (50/200) were nonambulatory; and for 10% (20/200), ambulation was not reported or unknown. In total, 39% (9/23) of the studies reported a measure of RTT severity, which included at least one participant with severe RTT. Only 22% (2/9) of these studies enrolled participants with mild RTT.

**Table 2:** Sample information (N=23).

Study	Year	Sample size, n	Mean age (y)	Age range (y)	Ambulatory	RTT^a^ severity	Waitlist control
Bumin et al [31]	2003	1	11	N/A^b^	1 yes	—^c^	—
Schaefer-Campion and Johnson [32]	2015	1	5	N/A	1 yes	—	—
Lerma-Castaño et al [33]	2024	1	10	N/A	1 yes	—	—
Downs et al [34]	2018	12	3	2-6	9 yes; 3 no	—	Crossover design
Downs et al [35]	2023	39	20	6-41	39 yes; 0 no	—	Control group
Imamura et al [36]	2020	1	17	N/A	1 yes	—	N/A
Kapel et al [18]	2022	5	31	17-44	3 yes; 2 no	—	N/A
Lotan et al [37]	2004	4	10	8-11	4 yes	—	Multiple baseline
Lotan and Barmatz [38]	2009	1	5	N/A	1 no	—	—
Lotan et al [39]	2015	1	—	N/A	1 yes	—	—
Lotan et al [40]^d^	2021	42	15.7	2-41	21 yes; 21 no	Moderate to severe	Multiple baseline
Lotan et al [41]	2021	5	9	5-18	4 yes; 1 no	—	—
Panzeri et al [42]	2024	9	8.6	6-44	9 yes	Moderate to severe	—
Rocco et al [43]	2023	1	8	N/A	1 yes	—	—
Romano et al [44]^d^	2022	42	15.7	2-41	21 yes; 21 no	Moderate to severe	Multiple baseline
Romano et al [45]	2022	20	15.6	3-39	Unknown	Moderate to severe	
Romano et al [46]	2022	13	17.9	3-35	8 yes; 5 no	Mild to severe	Multiple baseline
Romano et al [47]	2025	20	11.5	6-16	9 yes; 11 no	Mild to severe	Multiple baseline
Stahlhut et al [48]	2020	14	18.7	5-48	9 yes; 5 no	Moderate to severe	Multiple baseline
Stasolla and Caffo [49]	2013	2	9.5	2-17	2 yes	—	Multiple baseline
Stasolla et al [50]	2018	5	15	13-17	5 yes	Severe	Multiple baseline
Escobar Torres et al [51]	2019	3	Unknown	4-7	2 yes; 1 no	—	—
Zwilling et al [52]^d^	2022	42	15.7	2-41	21 yes; 21 no	Moderate to severe	Multiple baseline

^a^RTT: Rett syndrome.

^b^N/A: not applicable.

^c^Indicates staggered introduction of the intervention to establish experimental control.

^d^Indicates overlap in publications stemming from a single sample.

### Intervention Characteristics

The intervention characteristics are shown in Table 3. The mean intervention duration was 30 (SD 35) weeks, ranging from a single week to 3 years. The most common setting was in the home and community, followed by physical therapy clinics. Most interventions were delivered by health professionals directly or indirectly as an activity program designed for a caregiver or parent to deliver. Interventions were delivered from twice per week to daily, with the typical frequency being 5 times per week. Mean session duration was 55 (SD 36) minutes per week, with some durations as short as 5 to 10 minutes. The types of interventions varied. Some studies (20/23, 87%) included therapy-driven programs, hydrotherapy, walking, exercise training, and motor activities. While most studies (19/23, 83%) integrated a single type of intervention, some (4/23, 17%) were multimodal. For example, one study included 5 different intervention types (motor activities, hippotherapy, hydrotherapy, exercise training, and walking) [18]. While intervention characteristics were typically reported in detail (duration, setting, and delivery), protocols were often superficially described and lacked sufficient details to allow for replication. For example, frequency was often reported as recommended times per week without providing actual adherence. Additionally, time per session was typically reported as a range that could vary based on progression or unknown factors. Finally, most interventions relied on tailored, individualized exercise programs that listed the prescribed activities but did not provide specific dose information or movement adaptations.

**Table 3:** Intervention characteristics.

Study	Year	Intervention length	Setting	Delivery	Frequency (d per wk)	Duration (min per session)	Modality (activity)	Assessment
Bumin et al [31]	2003	8 wk	Swimming pool	Therapist	2	Unknown	Hydrotherapy (Halliwick concept)	Baseline and after the intervention
Schaefer-Campion and Johnson [32]	2015	24 wk	Elementary school	Special education physiotherapist	4	10 to 15	Walking trials with various assistive devices	—^a^
Lerma-Castaño et al [33]	2024	12 mo	Clinic	Physical therapist and behavioral therapist	3	45	PNF^b^, exercise activities, behavioral therapy, and physiotherapy	Baseline and after the intervention
Downs et al [34]	2018	6 mo	Clinic	Physical therapist	6	120 to 180	Motor activity with environmental stimulation	Baseline, after the intervention, 2-mo follow-up, and 4-mo follow-up
Downs et al [35]	2023	12 wk	Home, community, and school based	Caregiver	4	Variable	Standing and walking activities	Baseline and after the intervention
Imamura et al [36]	2020	4 mo	Clinic (weekdays) and home and community (weekends)	Physical therapist (weekdays) and caregiver (weekends)	7	40 at the clinic and 80 at home and in the community	Physical therapy, occupational therapy (clinic), and walking (community)	Each weekend day (MVPA^c^ and steps) and completion of first and final week of each intervention month (10-m walk test)
Kapel et al [18]	2022	12 mo	Various	Physical therapists	7	30 to 60	Neurodevelopmental, hippotherapy, hydrotherapy, exercise training, and walking	Baseline and after the intervention
Lotan et al [37]	2004	8 wk	Educational facility	National service person	7	5 to 30	Treadmill walking	Baseline, before the intervention, and after the intervention
Lotan and Barmatz [38]	2009	3 y	Clinic pool	Physical therapist	Unknown	Unknown	Hydrotherapy	Summation of reports for years 1, 2, and 3
Lotan et al [39]	2015	3 mo	Residential care facility	Caregiver	7	Unknown	Walking program enhanced with ABA^d^	Baseline and every session
Lotan et al [40]	2021	12 wk	Home based	Caregiver	5	60	Active and passive exercise and functional motoring activity	Baseline, before the intervention, after the intervention, and 3-mo follow-up
Lotan et al [41]	2021	6 mo	Home based	Caregiver	Unknown	Unknown	Physical therapy	Baseline and after the intervention
Panzeri et al [42]	2024	1 wk	Laboratory	Physical therapist	4	Unknown	Treadmill walking with semi-immersive virtual reality (GRAIL)	Baseline and every visit
Rocco et al [43]	2023	12 wk	Clinic	Physical therapist	3	60	Motor play activity with Ayres Sensory Integration	Every session
Romano et al [44]	2022	3 mo	Home based	Caregiver	5	60	Active and passive exercise and functional motoring activity	Baseline, before the intervention, after the intervention, and 3-mo follow-up
Romano et al [45]	2022	6 mo	Home based	Caregiver	5	60	Motor activities and postural program	Baseline and after the intervention
Romano et al [46]	2022	3 mo	Home based	Caregiver	5	60	Motor activities	Baseline, before the intervention, after the intervention, and 3-mo follow-up
Romano et al [47]	2025	10 mo	Home based	Caregiver	5	60	Postural and rehabilitation program	Baseline, before the intervention, and after the intervention
Stahlhut et al [48]	2020	12 wk	Home and community based	Caregiver	7	Unknown	Standing and walking activities	Baseline, before the intervention, after the intervention, and 3-mo follow-up
Stasolla and Caffo [49]	2013	6 mo	Home based	Research assistants	4	60	Microswitch-based program	Every session
Stasolla et al [50]	2018	3 mo	Rehabilitation facility	Research assistants	5	60 to 90	Microswitch-based program	Every session
Escobar Torres et al [51]	2019	20 mo	Community pool	Trainer	3	30	Hydrotherapy (WaterFit MITAF^e^ program)	Beginning of years 1 and 2 (functional performance) and end of year 1 (physical)
Zwilling et al [52]	2022	12 wk	Home based	Caregiver	5	60	Motor activity program	Baseline, before the intervention, after the intervention, and 3-mo follow-up

^a^Not applicable.

^b^PNF: Proprioceptive Neuromuscular Facilitation.

^c^MVPA: moderate to vigorous physical activity.

^d^ABA: Applied Behavior Analysis.

^e^MITAF: Integral Method of Functional Aquatic Work.

### Measured Outcomes

The primary and secondary outcomes and measures reported in the included studies are shown in Table 4, and frequencies are shown in Table 5. The most frequent primary or secondary outcome was gross motor function (11/23, 48%), followed by LTPA (6/23, 26%) or walking ability (6/23, 26%). Several of the studies (7/23, 30%) assessed behavior, goal attainment, caregiver satisfaction and well-being, fine motor function, or communication as a primary or secondary outcome. Health-related fitness outcomes were not measured thoroughly. Some studies (9/23, 39%) reported body composition, balance, quality of life, flexibility, cardiorespiratory health, intervention feasibility, or mood; however, these variables were typically secondary or tertiary measures. Additionally, very few objective measures were used to support the primary or secondary objectives, with most of the studies (21/23, 91%) relying on clinical observational performance or self-report (parent or caregiver) measures. There were no studies that reported direct measures of strength or cardiorespiratory health.

**Table 4:** Primary and secondary outcomes and measures.

Study	Year	Primary outcomes	Secondary outcomes	Measures
Bumin et al [31]	2003	Stereotypical hand movement, hand function, and feeding skills	Gait, balance, hyperactive behavior, communication, and social interaction	Observation
Schaefer-Campion and Johnson [32]	2015	Walking ability	Reason for discontinuing use	Initiation of walking, distance walked, and observation
Lerma-Castaño et al [33]	2024	Gross motor function	Behavior	EAD-3^a^ and behavioral observation scale
Downs et al [34]	2018	Gross motor function	BDNF^b^	RSGMS^c^, BMI, sleep disturbance scale for children, RTT^d^ behavior questionnaire (mood subscale), height, and blood level of BDNF protein
Downs et al [35]	2023	Sedentary time and number of steps	QOL^e^ and behavior	Accelerometer, step count, Quality of Life Inventory–Disability, sleep disturbance scale, and Mood and fear/anxiety subscales of the Rett Syndrome Behavior Questionnaire
Imamura et al [36]	2020	Time in MVPA^f^	Steps	Step and activity counts and 10-m walk test
Kapel et al [18]	2022	Gross motor function	N/A^g^	RSGMS and Gross Motor Function Measure–88
Lotan et al [37]	2004	Cardiorespiratory fitness	Gross motor function	RHR^h^ to peak HR^i^ and motor function scale (researcher made)
Lotan and Barmatz [38]	2009	Gross motor function	Communication and behavior	Observation
Lotan et al [39]	2015	Daily steps	—^j^	Accelerometer
Lotan et al [40]	2021	Parental satisfaction	Goal attainment and gross motor function	GAS^k^, questionnaire, and semistructured interview
Lotan et al [41]	2021	Feasibility	Goal attainment and parental satisfaction	GAS and satisfaction survey
Panzeri et al [42]	2024	Feasibility	Suitability, happiness, endurance, speed, and attention focus	Happiness index, time played until discomfort, peak treadmill speed, observation, and SEQ^l^
Rocco et al [43]	2023	Barriers to and facilitators of participation	N/A	Observation
Romano et al [44]	2022	Goal attainment and gross motor function	PA^m^ level	GAS, RESMES^n^, and mBAR^o^
Romano et al [45]	2022	Scoliosis severity	PA level and gross motor function	Clinical scoliosis score, mBAR, and RESMES
Romano et al [46]	2022	Gross motor function and goal attainment	Caregiver satisfaction, scoliosis severity, and ROM^p^	GAS, RESMES, joint angle measurement, clinical scoliosis score, and caregiver survey
Romano et al [47]	2025	Scoliosis severity	Gross motor function	Clinical scoliosis score and RESMES
Stahlhut et al [48]	2020	Feasibility, sedentary time, and daily PA	Gross motor skills, walking capacity, and QOL	Step and activity counts, caregiver activity diary, RSGMS, 2-min walk test, and QOL—disability
Stasolla and Caffo [49]	2013	Walking ability	Happiness and stereotyped behavior	Total microswitch activations, indexes of happiness (observed), and observation
Stasolla et al [50]	2018	Walking ability	Happiness, stereotyped behavior, and social validation	Total microswitch activations, indexes of happiness (observed), and observation
Escobar Torres et al [51]	2019	Resting HR and exercise HR	Body composition, joint mobility, ADLs^q^, and functional independence	HR, skinfolds, Barthel ADL index, functional independence measure, and clinical and psychological evaluation
Zwilling et al [52]	2022	Mothers’ well-being	N/A	Caregiver well-being scale

^a^EAD-3: Evaluation of Aquatic Abilities–Third Edition.

^b^BDNF: brain-derived neurotropic factor.

^c^RSGMS: Rett Syndrome Gross Motor Scale.

^d^RTT: Rett syndrome.

^e^QOL: quality of life.

^f^MVPA: moderate to vigorous physical activity.

^g^N/A: not applicable.

^h^RHR: resting heart rate.

^i^HR: heart rate.

^j^Not available.

^k^GAS: Goal Attainment Scaling.

^l^SEQ: Suitability Evaluation Questionnaire.

^m^PA: physical activity.

^n^RESMES: Rett Syndrome Motor Evaluation Scale.

^o^mBAR: modified Bouchard activity record.

^p^ROM: range of motion.

^q^ADL: activity of daily living.

**Table 5:** Frequency of outcome measures across included studies (n=23).

Outcome measure	Frequency, n (%)
Gross motor function	11 (47.8)
Physical activity	6 (26.1)
Walking ability	6 (26.1)
Behavior	5 (21.7)
Caregiver satisfaction and well-being	5 (21.7)
Fine motor function	4 (17.4)
Goal attainment	4 (17.4)
Communication	3 (13)
Mood	2 (8.7)
Feasibility	2 (8.7)
Cardiorespiratory health	2 (8.7)
Flexibility	2 (8.7)
Quality of life	2 (8.7)
Balance	1 (4.3)
Body composition	1 (4.3)

The studies that reported gross motor function were split between the use of RTT-specific motor assessment tools, including the Rett Syndrome Gross Motor Scale [53] and the Rett Syndrome Motor Evaluation Scale [54]. Physical activity was typically measured through step or activity counts using an accelerometer; however, 9% (2/23) of the studies estimated LTPA using a modified Bouchard activity record [55].

### Telehealth Implementation

Among the 23 studies included in this review, 9 (39%) interventions were delivered through telehealth. These studies accounted for 77% (153/200) of the total participant population. Ambulatory participants made up approximately 59% of the total telehealth enrollment. Every telehealth study used a remote video call delivered by a health professional to a caregiver. Only one study augmented the video calls with an online monitoring platform (smartphone app) [47]. The most common frequency of video calls was once every other week (67%). Several studies only used video calls as needed or monthly. Very few telehealth procedural details (eg, communication, behavior change, or technical implementation strategies) were reported across these studies to support replication.

## Discussion

### State of Research

This scoping review summarized 23 studies involving LTPA for individuals with RTT, with a secondary aim of charting telehealth implementation. The findings reveal a growing body of evidence supporting the feasibility and potential benefits of LTPA interventions across diverse settings, delivery modes, and outcome domains. We discuss the current state of research on LTPA interventions, telehealth implementation, expected benefits, and recommendations for future research that could enhance LTPA interventions for individuals with RTT.

These study findings, similar to those of a recent scoping review of rehabilitation therapies, suggest a lack of studies that could be classified as high-quality evidence [56]. Regarding quality, there appeared to be 2 key limitations: sample size and study design. While many global regions were represented in our review (Table 1), study samples were predominantly small. Over half (12/23, 52%) of the studies included in this review did not incorporate a control or comparison group, and many studies (9/23, 39%) instead used a multiple-baseline design in which the intervention was introduced at different times across participants, settings, or behaviors to demonstrate experimental control (Table 2). Single-group within-subject designs may be appropriate for evaluating the effects of LTPA interventions in neuromuscular disorders, particularly in the context of small sample sizes (eg, 10-40 participants) [57]. On a positive note, our findings suggest that several research design decisions across these studies were consistent with recommendations from the International Rare Diseases Research Consortium for small-population clinical trials, including making use of longitudinal data to show how treatment effects evolve over time, using multiple end points, adjusting follow-up outcomes for baseline values rather than relying on a single change-from-baseline measure, and using registry data to aid in sample size estimates [58]. The only RCTs (2/23, 9%) were implemented by one researcher [34,35]. These studies were able to demonstrate an increase in gross motor skills with environmental enrichment and a small reduction in sedentary time through a telehealth-supported program.

Disappointingly, low rates of physical activity are common in RTT, particularly among people with RTT who have a mobility disability and a higher age [59,60]. A notable finding was that several types of home- and community-based LTPA interventions were successful in achieving their intended outcomes. An encouraging finding was that these interventions were adapted to the needs of individuals with RTT who were ambulatory and nonambulatory. Nonambulatory participants represented 65% of the sample, which was representative of the population [61]. Such adaptations and programs were particularly noteworthy for their inclusion of individuals with more severe levels of disability. However, the lack of depth in reporting intervention and implementation characteristics limits replication of current research efforts in this area. We encourage future LTPA research in RTT to provide more transparent details regarding intervention tailoring strategies, along with successful and unsuccessful lessons that have been learned from intervention delivery.

We found that caregivers were often the primary target for LTPA interventions. This approach likely reflects the severity of the children’s disability, which limits the feasibility of direct participation or communication. Although caregivers play a vital role in fostering LTPA, particularly when communication difficulty in the child is a concern, caregivers experience substantial burden with LTPA interventions, which can manifest in various barriers, including limited time and resources, difficulty obtaining mobility aids, few trained professionals, and a lack of knowledge [62]. Caregivers of children or adult patients with RTT report barriers to LTPA that are consistent with those of other disability groups [62,63], including limited resources in the community, lack of transportation, lack of trained professionals, lack of suitable LTPA modalities and resources, and lack of knowledge [64,65]. Therefore, LTPA programs that are accessible and adaptable to the unique needs of individuals with RTT may promote LTPA and reduce caregiver burden [4].

Many of the targeted outcomes reported across the studies in this review were not objectively measured. There was a lack of health and fitness outcomes that are typical in LTPA interventions. For instance, there was not a single example of an objective muscle strength measure even though arm and leg muscle area measurements are significantly lower in female patients with RTT than in their male counterparts [3]. Gross motor function was a consistent positive finding [18,33,34]. There was little evidence to demonstrate an improvement in cardiopulmonary health, which is concerning because RTT is associated with cardiac arrhythmias that are a potential cause of sudden death [3]. Therefore, benefits as a result of LTPA for people with RTT should be interpreted with caution.

A notable gap identified in this study was the limited information on telehealth implementation. The use of telehealth as an intervention delivery platform may expand intervention reach and address enrollment barriers. Delivery of interventions through telehealth has been shown to be effective and important in neurodevelopmental rehabilitation and disability management [66,67]. Remote interventions in this review demonstrated positive goal achievement and caregiver satisfaction [35,40,41,47,48]. Consistent with findings across all included studies, we observed little to no direct communication with participants with RTT via remote telecommunication. While impaired communication is common in individuals with RTT [68], a major concern of their caregivers [69], and a barrier to LTPA participation [62], it is unclear whether direct participant communication through telehealth is feasible. For example, one study demonstrated significant potential in supplementing telehealth coaching using alternative augmentative communication [70]. While direct interaction with participants is typical in telehealth exercise interventions for individuals with disabilities [71], achieving meaningful communication with participants with RTT may require additional support from speech-language therapists or a family member. Although the intervention characteristics were often superficially reported across studies, the description of telehealth delivery and engagement of participants with RTT was even more limited. A deeper understanding of the factors contributing to telehealth implementation success and failure could help guide future research in this area.

### Consultation Exercise

The consultation exercise reinforced and contextualized several key gaps identified in the literature. The consulted caregiver emphasized that effective LTPA interventions for individuals with RTT must be tailored to age and ambulation status, echoing the heterogeneity observed across the included studies. Their insights highlighted the potential value of telehealth in supporting participation and accountability for families, particularly given the heavy caregiving demands that limit opportunities for structured physical activity. Importantly, the caregiver identified a critical service gap wherein adolescents and adults with RTT often lose access to outpatient therapy services, underscoring the need for accessible, home‑based, or remotely delivered programs. The consultation also drew attention to the lack of standardized severity classifications in existing research and the absence of key outcome domains—such as muscle strength, cardiovascular health, balance, and quality of life—that families consider essential for daily functioning. These perspectives align with the broad variability in intervention characteristics and outcome measures found in our review and further support the need for consistent reporting and comprehensive assessment frameworks. Collectively, the consultation emphasized the urgency of developing adaptable, evidence-informed LTPA resources and best practice guidelines across the life span, particularly for nonambulatory individuals and those aging out of pediatric services.

### Future Recommendations

The most promising outcomes observed in this scoping review were improvements in physical activity and walking ability. To advance the field and address existing gaps, future research should consider the following. First, studies should include more adults with RTT to better understand age-related changes in LTPA participation and intervention responsiveness across the life span. Second, while the feasibility of interventions was often reported as good, future research should add greater levels of detail in their protocols and modalities to increase the reproducibility of their design. For example, the walking trials administered by Schaefer-Campion and Johnson [32] illustrated nuanced differences in walking aids for a child with RTT that can inform decisions for future studies to foster walking ability. Conducting larger and more frequent multisite RCTs with appropriate control conditions may strengthen causal inferences. Adopting objective measures for aspects of health-related fitness such as strength across studies would facilitate cross-study comparisons and meta-analyses. Because low physical fitness, gait abnormalities, and comorbidities such as scoliosis and osteoporosis can be potential barriers to participation in LTPA [4,72,73], it is important to confirm the short-term efficacy of interventions that may mitigate barriers and increase LTPA in RTT.

Moreover, the addition of telehealth communications and remotely delivered interventions at home may be ideal for overcoming community barriers to LTPA among people with RTT. Telehealth LTPA clinical trials bypass the need for on-site visitation and have reached the largest sample sizes among people with physical disabilities, which would be ideal for rare diseases and conditions such as RTT [74,75].

### Limitations

Several limitations should be acknowledged. Many of the included studies partially reported or did not report at all critical variables such as RTT severity, exercise protocols, or outcome frequency, thereby making it difficult to generalize the findings of this scoping review. Due to the lack of randomized trials, this review focused on non-RCTs, and the heterogeneity in study designs, sample sizes, and intervention protocols limits the generalizability of the findings. Classifying interventions as LTPA was challenging because the studies often involved a mixture of community-based exercise and physical therapy. This limitation reflects the field itself rather than being a limitation of this review specifically. Different operational definitions of LTPA could yield different conclusions. The heterogeneity of RTT (eg, variability in severity, age, and functional ability), combined with small sample sizes and diverse study designs, limits comparability across studies and complicates the synthesis of findings and interpretation of overall effects. Reliance on observational measures and caregiver-reported outcomes introduces potential bias. Finally, with a strong focus on pediatric populations, this review highlights the lack of evidence related to adults with RTT despite over half of individuals with RTT living beyond 50 years of age [6].

### Conclusions

This review underscores the potential of LTPA interventions to enhance wellness and functional outcomes in individuals with RTT. Telehealth strategies show promise for improving accessibility and caregiver engagement. Nevertheless, further research is needed to establish best practices, optimize delivery models, and ensure inclusivity across the life span. Future studies should incorporate objectively measured health outcomes to verify both short- and long-term benefits while also providing detailed reporting on intervention tailoring and implementation strategies, including factors contributing to both successes and challenges.

## Appendix

Multimedia Appendix 1 PRISMA-ScR checklist.

## Abbreviations

LTPA: leisure-time physical activity
PRISMA: Preferred Reporting Items for Systematic Reviews and Meta-Analyses
PRISMA-ScR: Preferred Reporting Items for Systematic Reviews and Meta-Analyses Extension for Scoping Reviews
RCT: randomized controlled trial
RTT: Rett syndrome

## Data Availability

Data are available upon request to the corresponding author.
